# MyD88 Is Required for Efficient Control of *Coxiella burnetii* Infection and Dissemination

**DOI:** 10.3389/fimmu.2019.00165

**Published:** 2019-02-08

**Authors:** Lisa Kohl, Inaya Hayek, Christoph Daniel, Jan Schulze-Lührmann, Barbara Bodendorfer, Anja Lührmann, Roland Lang

**Affiliations:** ^1^Institute of Clinical Microbiology, Immunology and Hygiene, Universitätsklinikum Erlangen, Friedrich-Alexander-Universität Erlangen-Nürnberg, Erlangen, Germany; ^2^Department of Nephropathology, Institute of Pathology, Universitätsklinikum Erlangen, Friedrich-Alexander-Universität Erlangen-Nürnberg, Erlangen, Germany

**Keywords:** *Coxiella burnetii* nine mile phase II, Toll-Like Receptor (TLR), Q fever, mouse model, chronicity, resolution, intratracheal

## Abstract

The intracellular pathogen *Coxiella (C.) burnetii* causes Q fever, a usually self-limiting respiratory infection that becomes chronic and severe in some patients. Innate immune recognition of *C. burnetii* and its role in the decision between resolution and chronicity is not understood well. However, TLR2 is important for the response to *C. burnetii* in mice, and genetic polymorphisms in *Myd88* have been associated with chronic Q fever in humans. Here, we have employed MyD88-deficient mice in infection models with the attenuated *C. burnetii* Nine Mile phase II strain (NMII). *Myd88*^−/−^ macrophages failed to restrict the growth of NMII *in vitro*, and to upregulate production of the cytokines TNF, IL-6, and IL-10. Following intraperitoneal infection, NMII bacterial burden was significantly higher on day 5 and 20 in organs of *Myd88*^−/−^ mice. After infection *via* the natural route by intratracheal injection, a higher bacterial load in the lung and increased dissemination of NMII to other organs was observed in MyD88-deficient mice. While wild-type mice essentially cleared NMII on day 27 after intratracheal infection, it was still readily detectable on day 42 in multiple organs in the absence of MyD88. Despite the elevated bacterial load, *Myd88*^−/−^ mice had less granulomatous inflammation and expressed significantly lower levels of chemoattractants, inflammatory cytokines, and of several IFNγ-induced genes relevant for control of intracellular pathogens. Together, our results show that MyD88-dependent signaling is essential for early control of *C. burnetii* replication and to prevent systemic spreading. The continued presence of NMII in the organs of *Myd88*^−/−^ mice constitutes a new mouse model to study determinants of chronicity and resolution in Q fever.

## Introduction

The intracellular bacterium *Coxiella burnetii* is the pathogenic agent of the zoonotic disease Q fever, which usually presents as self-limiting respiratory tract infection after inhalation of aerosolized bacteria shed by infected small ruminants. In a small percentage of human patients, *C. burnetii* infection does not resolve, but develops into severe and chronic infection affecting the vasculature, including endocarditis. Host factors associated with an increased risk to develop chronic Q fever are older age, cardiac valve abnormalities, pregnancy and immunosuppression (1, 2).

Key immunological host factors required to control *C. burnetii* infection have been identified in the mouse model. Since *C. burnetii* resides and proliferates intracellularly, mostly in macrophages, it is not surprising that protective host immunity appears to rely on T cells and IFNγ, as is the case in other intracellular infections. The pivotal importance of T cells has been demonstrated in SCID mice and nude mice (3). Production of IFNγ, produced by Th1, CD8^+^ T cells or NK cells, is essential to control infection with *C. burnetii* in the murine system (3). By which mechanisms IFNγ signaling induces the killing of *C. burnetii* in macrophages is only incompletely understood, but involves the production of reactive nitrogen intermediates by iNOS, at least in the murine model (4). Interestingly, the production of IFNγ appears not to be deficient in patients with chronic Q fever (5), suggesting that other host factors are involved. The immunomodulatory cytokine IL-10 deactivates macrophages through Stat3-dependent signaling, leading to impaired production of cytokines like TNF and IL-12 (6). IL-10 is overproduced by monocytes of patients with chronic Q fever (7) and impairs killing of *C. burnetii* in human macrophages (8). In addition, mice overproducing IL-10 from macrophages (9) have higher and prolonged bacterial burden after infection with *C. burnetii* (10), constituting a mouse model for chronic Q fever.

Impaired sensing of *C. burnetii* by the innate immune system may be another explanation for the development of chronic infection in some patients. This notion is in fact supported by the demonstration that a single nucleotide polymorphism in the Toll-like receptor (TLR) adapter protein MyD88 was associated with development of chronic Q fever in a large cohort of Dutch patients (11). A role for TLR2 as pattern recognition receptor for *C. burnetii* was already established in 2004 in mouse macrophages (12) and has been confirmed in human cells (13). MyD88 was recently demonstrated to be required for induction of TNF production and control of bacterial replication in murine macrophages infected with *C. burnetii in vitro* (14). Furthermore, TLR2- and MyD88-deficient mice developed increased bacterial burden after intratracheal infection with *C. burnetii* (15).

*Coxiella burnetii* shows phase variation with regard to LPS synthesis. Phase I *C. burnetii* synthesizes LPS with a highly branched O-chain, which has been traditionally considered the major virulence factor because it is the form isolated from patients with Q fever (16). Serial *in vitro* culture resulted in a shift to phase II LPS variants with truncated O-antigen polysaccharides, which in the case of the *C. burnetii* Nine Mile phase II clonal derivative is due to a chromosomal 26 kB deletion affecting several LPS biosynthesis genes (17). Since the *C. burnetii* Nine Mile Phase 2 RSA 439 clone 4 (NMII) was found to be less virulent than the phase I parent strain in immunocompetent mice and guinea pigs (18, 19), it can be used under Biosafety Level 2 conditions. Importantly, both phase variants show similar growth in a modified phagosome, the *Coxiella*-containing vacuole of murine and human macrophages (20, 21). Furthermore, recent studies have clearly shown that the attenuated NMII strain is virulent in SCID mice that lack T and B lymphocytes (22, 23), demonstrating that virulence of *C. burnetii* cannot be reduced to phase I LPS, and providing an opportunity to study bacterial and host factors that determine the course of infection in a more amenable mouse model.

Here, we have used MyD88-deficient mice to investigate the course of infection with the attenuated NMII strain. We found that MyD88 was required in macrophages *in vitro* for restricting growth of NMII and for expression and secretion of cytokines. *Myd88*^−/−^ mice developed higher bacterial loads in spleen, liver and lung after intraperitoneal infection. This phenotype was confirmed after intratracheal infection with NMII, where an essential role of MyD88 in efficient elimination of NMII from the lung and from other tissues, including the heart, was observed. In the absence of MyD88, mice had reduced granulomatous inflammation and showed impaired expression of proinflammatory cytokines and chemokines, as well as a reduced IFNγ-response. Infection of *Myd88*^−/−^ mice with the attenuated NMII strain proved to model important aspects of chronic Q fever (prolonged *C. burnetii* load in several tissues; impaired granuloma formation; and altered cytokine expression). We therefore propose this experimental setting as a promising model to further explore the contribution of host factors, such as IFNγ-induced genes, but also of bacterial factors, to the control of infection and resolution of inflammation.

## Results

### NMII Replicates in MyD88-Deficient BMM

**Figure 1:**
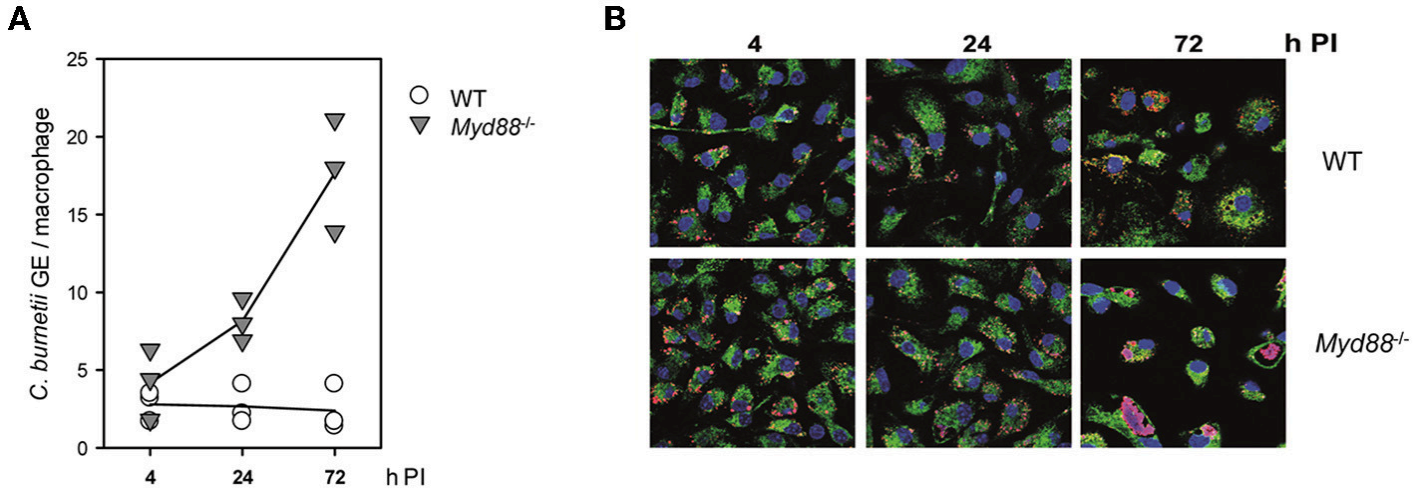
Replication of NMII in MyD88-deficient macrophages. MyD88-deficient (*Myd88*^−/−^) and wild-type BMM (WT) were infected with NMII (MOI of 10). **(A)** At the indicated time points post-infection (PI), bacterial load was assessed by qPCR for the *C. burnetii* dotA gene and the murine albumin gene. Results of three independent experiments are shown. **(B)** BMM were infected on glass coverslips. The cells were fixed and stained at the indicated time points with DAPI (blue) and with antibodies against LAMP1 (green) and *C. burnetii* (red). One of 3 experiments with similar results is shown.

We first tested the role of MyD88 signaling in the interaction of *C. burnetii* Nine Mile phase II (NMII) with macrophages *in vitro*. Bone marrow-derived macrophages (BMM) from *Myd88*^−/−^ and *Myd88*^+/−^ or C57BL/6 control mice were infected and analyzed over time for the growth or persistence of NMII by qPCR for the bacterial *dotA* gene and the murine *albumin* gene (Figure 1A) and immunofluorescence detection of NMII (Figure 1B). Early after infection, at 4 h, comparable levels of NMII genome equivalents (GE) per macrophage were found by qPCR, indicating that MyD88 is not controlling the uptake or early killing of NMII. While wild type macrophages controlled NMII, with moderately decreasing signals detected by qPCR and by immunofluorescence after 24 and 72 h, the NMII GE number per macrophage already increased after 24 h and continued to do so after 72 h, reaching a roughly five-fold higher level compared to the 4 h time point. Immunofluorescence staining of NMII revealed the development of large *Coxiella*-containing vacuoles (CCV) in a quite large fraction of *Myd88*^−/−^ BMM after 72 h, whereas the size of the CCV did not augment in wild type BMM (Figure 1B). Thus, MyD88 was not involved in the uptake of NMII, but was essential to inhibit bacterial replication and development of a large CCV.

### Differential Requirement for MyD88 in Cytokine Production and Enzyme Expression by NMII-infected Macrophages

**Figure 2:**
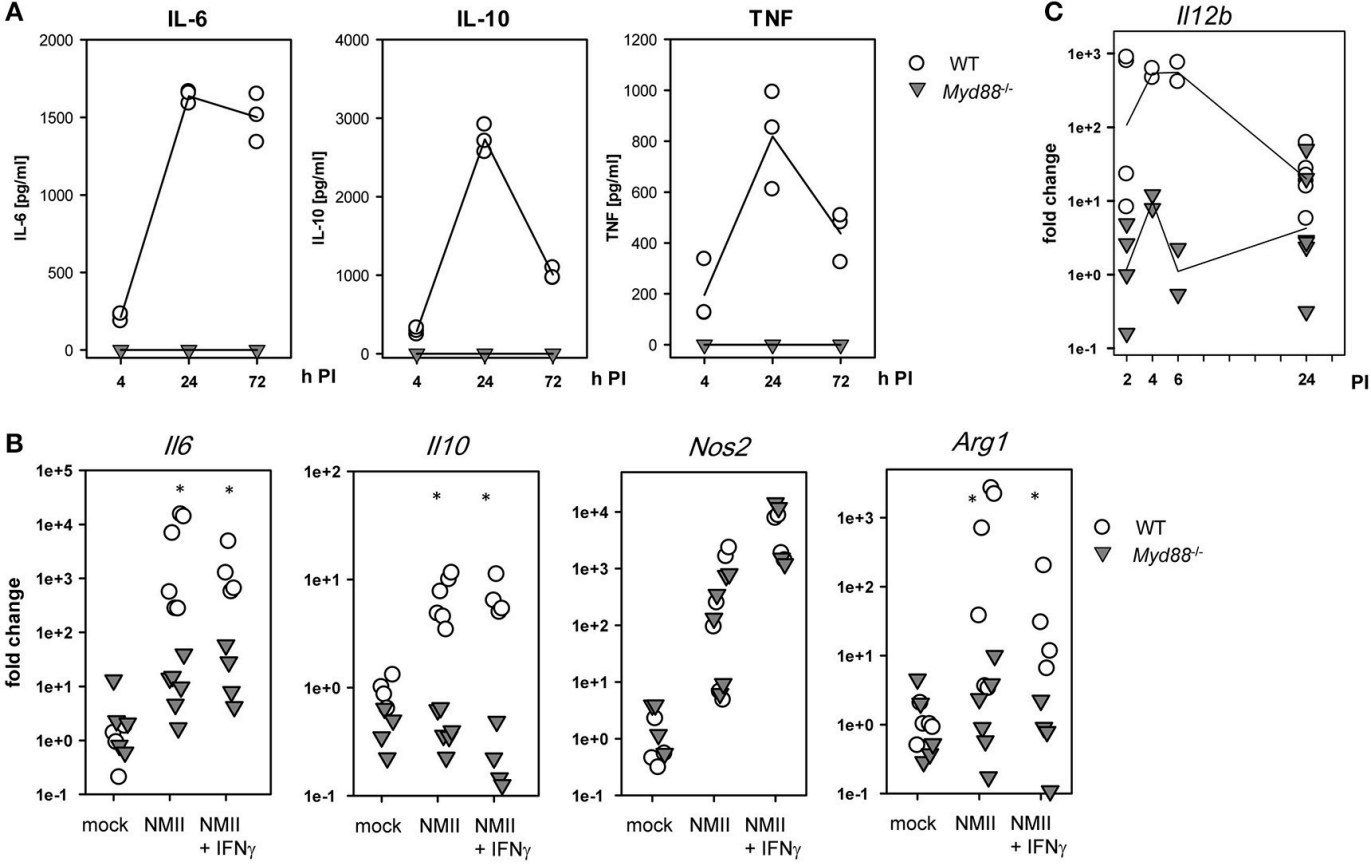
Dependence of *C. burnetii*-induced cytokine and enzyme expression on MyD88 in BMM. **(A)** supernatants of BMM infected with NMII at an MOI of 10 were used to determine secreted cytokine levels by ELISA of IL-6, IL-10, and TNF. Mean values of duplicate wells from three independent experiments are shown. **(B)** BMM were plated in antibiotic-free cDMEM overnight. NMII was added to the BMM (MOI 10) in the presence or absence of IFNγ (10 ng/ml). After 3 h, extracellular NMII was washed away. Cells were lysed after 24 h for preparation of RNA. Relative quantitation was performed by ΔΔCT method, using the mean ΔCT of non-infected BMM as calibrator. Pooled data from two (NMII+IFNγ) and three experiments (mock / NMII) performed with biological duplicates are shown. Mann-Whitney Rank Sum Test, asterisks indicate *p* < 0.05. **(C)** kinetic analysis of *Il12b* mRNA expression. RNA was harvested form BMM at the indicated times after infection with NMII (MOI 10) and processed for qRT-PCR. Pooled data from 3 experiments, each dot represents RNA from a biological replicate.

The production of TNF, IL-6, and IL-10 by BMM infected with NMII was robustly detectable in supernatants of wildtype BMM and completely dependent on MyD88 (Figure 2A). Measurement of mRNA induction by qRT-PCR confirmed this effect and dependence on MyD88 for IL-6 and IL-10 (Figure 2B). Of interest, induced expression of INOS, a critical anti-microbial enzyme involved in control of *C. burnetii* (4, 24), was induced to the same level in BMM from *Myd88*^−/−^ as wild-type mice. Upregulation of the other arginine-utilizing enzyme in macrophages, Arginase-1, by NMII was again completely abrogated in *Myd88*^−/−^ BMM. Expression of *Il12b* mRNA was strongly and MyD88-dependently induced by NMII after 2–6 h, and down-regulated after 24 h (Figure 2C). Together, these data identified a critical role for MyD88 in the induction of important pro- and anti-inflammatory cytokines in response to NMII infection *in vitro*, but also suggest that not all transcriptional responses in NMII-infected BMM require MyD88 signaling.

### Increased NMII Burden in Spleen, Liver and Lung After Intraperitoneal Infection

**Figure 3:**
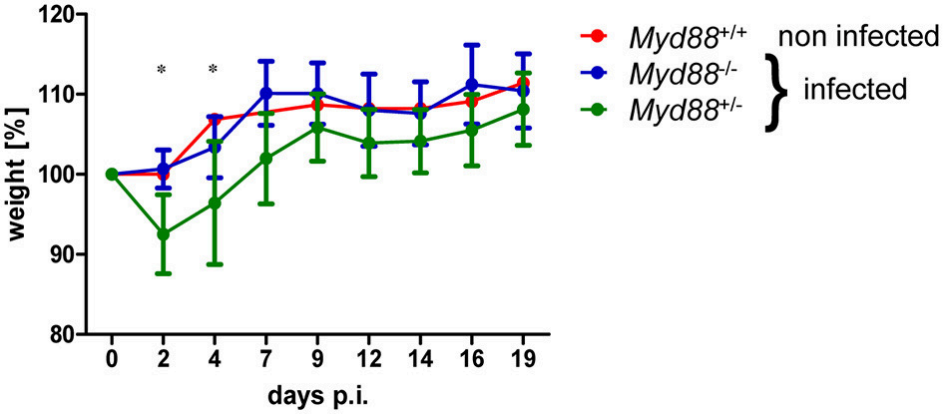
Transient weight loss after NMII infection. Mice were weighed before and at indicated time points after intraperitoneal infection with 5 × 10^7^ NMII. Changes in weight relative to day 0 are shown (mean ^+/−^ SD). One non-infected *Myd88*^+/+^ mouse was included for comparison. (*n* = 8 (day 0–5) and *n* = 4 (day 6–20) for *Myd88*^+/−^), *n* = 7 (day 0–5) and *n* = 3 (d6–20) for *Myd88*^−/−^). Results were analyzed using an unpaired two-tailed Mann-Whitney *U* test.

**Figure 4:**
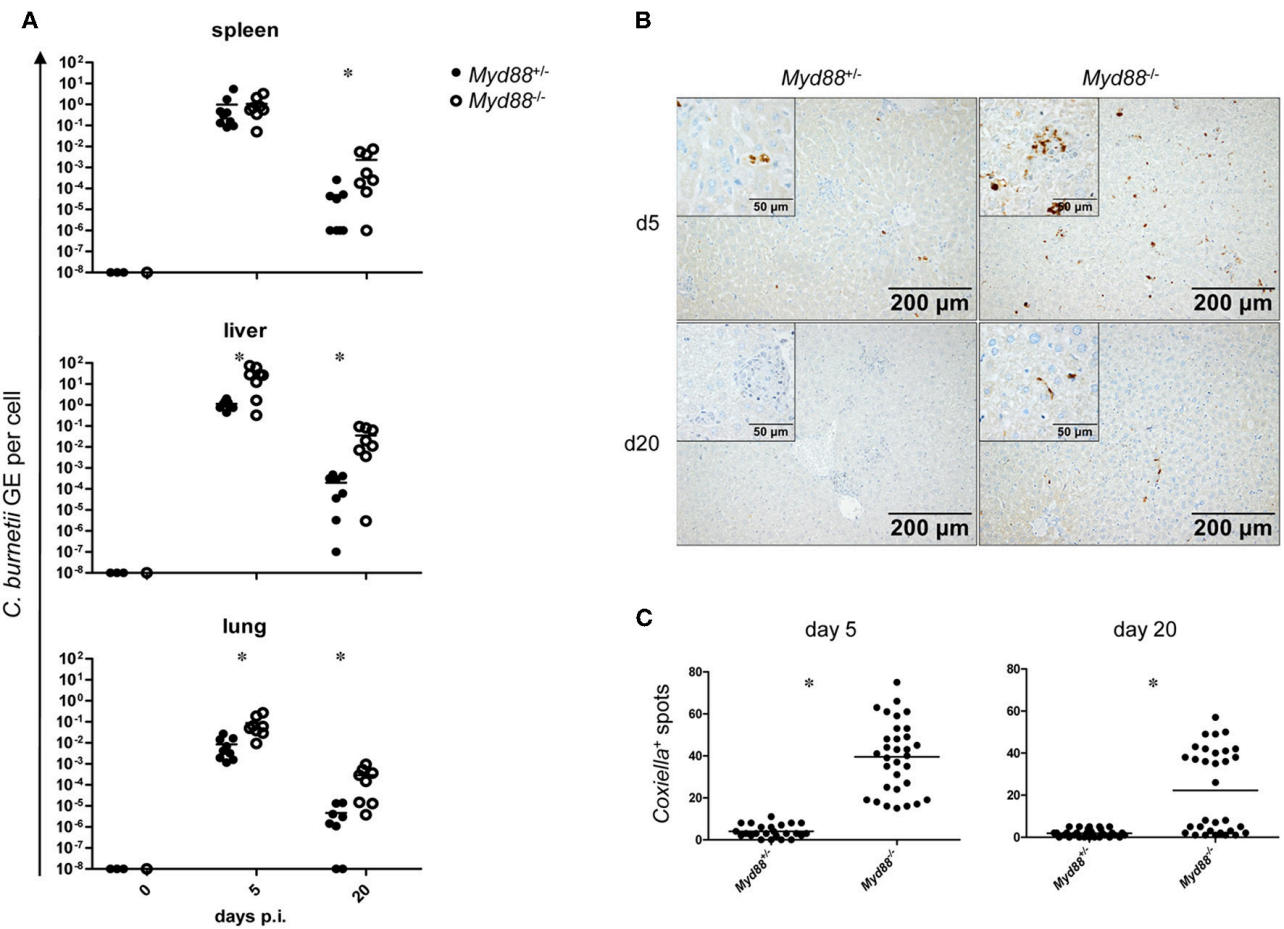
Increased bacterial load in *Myd88*^−/−^ mice after intraperitoneal infection. Mice were infected i.p. with 5 × 10^7^ CFU NMII and sacrificed after 5 and 20 days. **(A)** bacterial burden in spleen, liver and lung was assessed by qPCR for the *C. burnetii* insertion sequence IS1111 and for the murine albumin gene. Shown are NMII GE per cell. Each dot in the graphs represents one single mouse; data were pooled from two independent experiment. **(B)** immunohistochemical staining of liver sections with anti-*C. burnetii* antiserum shown at 100x magnification. (Inset) enlarged view at 400x magnification. **(C)** quantitation of *Coxiella*-containing cells (dark spots) per field of view at 100x magnification was performed for eight randomly chosen positions on sections from 4 individual mice per condition. Results were analyzed using an unpaired two-tailed Mann-Whitney *U* test.

To determine whether MyD88-dependent signaling is required for control of NMII *in vivo*, mice were first infected by the intraperitoneal route with 5 × 10^7^ NMII. A moderate but significant decrease in body weight was observed in *Myd88*^+/−^ mice on day 2 after infection, but not in *Myd88*^−/−^ mice (Figure 3). However, both *Myd88*^+/−^ and *Myd88*^−/−^ mice did not show signs of disease, appeared normal following infection and gained weight at a similar rate after 4 days of infection. The bacterial burden in spleen, liver and lung was analyzed by qPCR detection of the *C. burnetii* insertion sequence IS1111 as genome equivalents (GE) per cell on day 5 and 20 after i.p. infection (Figure 4A). NMII GE on day 5 were comparable between genotypes in the spleen but significantly higher in liver and lung of *Myd88*^−/−^ mice. On day 20, the NMII load was already reduced by 4 orders of magnitude compared to day 5 levels in *Myd88*^+/−^ organs, indicating efficient and ongoing elimination of bacteria. In contrast, bacterial load decreased less efficiently in *Myd88*^−/−^ organs, where the NMII GE were significantly higher than in *Myd88*^+/−^ spleen, liver and lung. To complement quantitative PCR measurement of NMII genomes by an independent method showing the spatial distribution of bacteria in the tissue, we employed immunohistochemical detection of NMII in liver tissue at both time points (Figures 4B,C). Consistent with the higher NMII GE detected in the *Myd88*^−/−^ liver already at day 5, we observed more *Coxiella*-positive spots in the *Myd88*^−/−^ liver sections. This difference was again more pronounced at day 20, when most liver sections of *Myd88*^+/−^ mice were negative for immunohistochemical staining of *C. burnetii*, but *Myd88*^−/−^ livers still contained easily detectable bacteria. Thus, both detection of NMII GE by qPCR and immunohistochemical staining of NMII consistently showed significantly higher bacterial burden in *Myd88*^−/−^ organs after intraperitoneal infection, which was more pronounced at the later time point (day 20) after infection. Nevertheless, also *Myd88*^−/−^ mice could reduce bacterial burden between day 5 and 20, albeit less efficiently than *Myd88*^+/−^ controls.

### Increased Dissemination and Persistence of NMII in Myd88^−/−^ Mice After Intratracheal Infection

**Figure 5:**
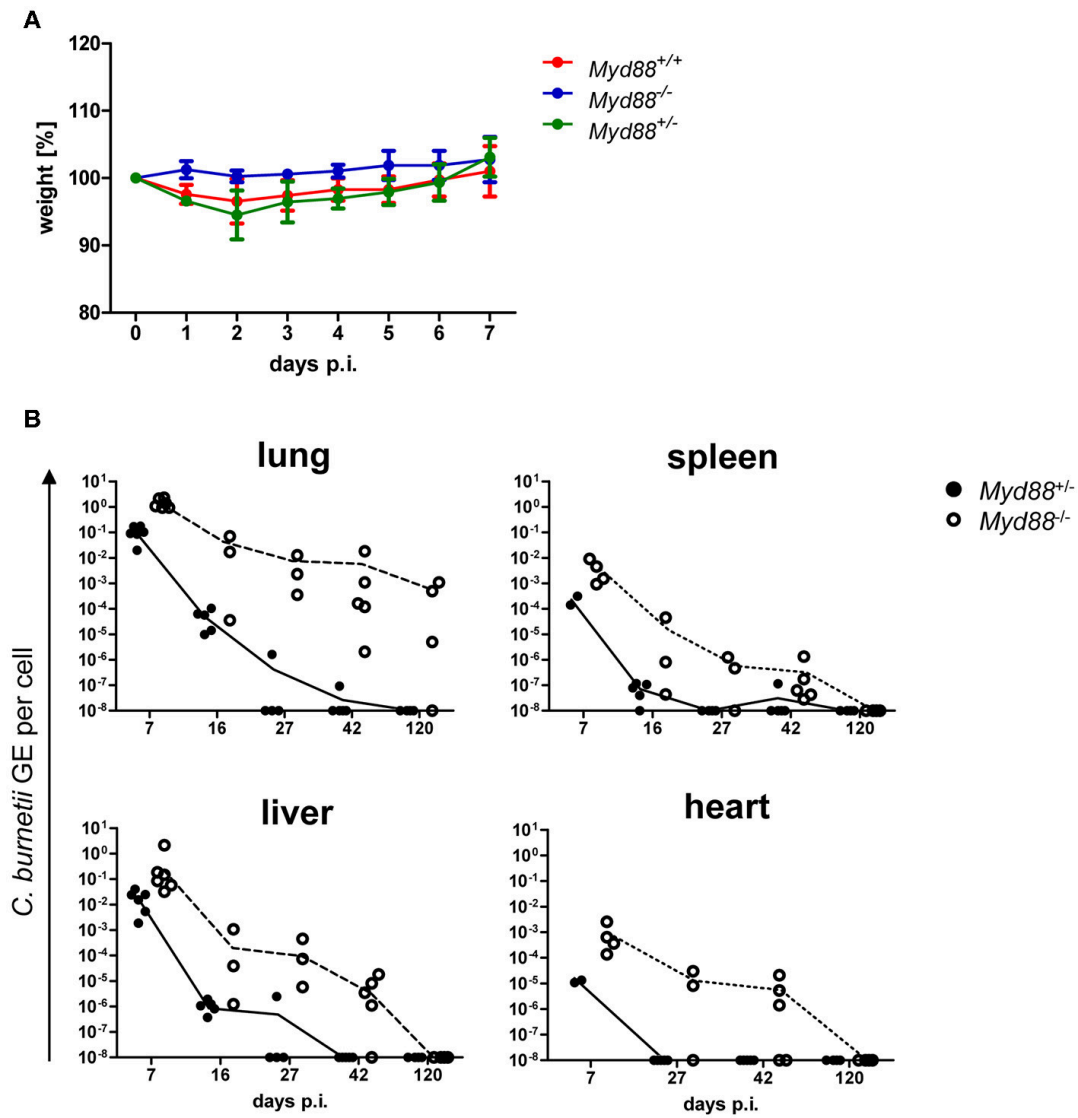
Kinetics of *C. burnetii* persistence after intratracheal infection. Intratracheal infection of mice was performed with 10^6^ CFU *C. burnetii* NMII. **(A)** The weight of the mice was recorded daily for 7 days and changes relative to d0 are depicted as mean ^+/−^ SD (*n* = 4 for *Myd88*^+/+^ and for *Myd88*^+/−^, *n* = 2 for *Myd88*^−/−^). **(B)** mice were sacrificed on day 7, 16, 27, 42, or 120 post infection. Bacterial load in lung, spleen, liver and heart was determined via qPCR as NMII GE per cell. Every single dot in the graphs represents one single mouse.

The natural route of infection with *C. burnetii* is *via* the airways by aerosolized bacteria. Recently, a higher permissiveness of alveolar macrophages for *C. burnetii* replication has been reported for *in vitro* as well as *in vivo* infection (25, 26). Therefore, we infected mice by intratracheal injection with 10^6^ NMII and determined weight loss as sign of clinical disease and the bacterial load as GE by qPCR in different organs over time (Figure 5). Similar to what was observed after intraperitoneal infection, *Myd88*^+/−^ and *Myd88*^+/+^ mice experienced a transient and moderate loss in weight with a nadir at d2 post intratracheal infection, but regained weight within 1 week (Figure 5A). In contrast, MyD88-deficient mice were protected from weight loss after intratracheal infection. Both *Myd88*^−/−^ and *Myd88*^+/−^ mice did not show signs of clinical disease during the further course of the experiment (d7 to d120). As expected, the highest NMII GE were found in the lung at the early time point on day 7. While *Myd88*^+/−^ mice efficiently reduced NMII in the lungs already at day 16 and eliminated the bacteria before day 42, in *Myd88*^−/−^ mice NMII GE were already higher on day 7, and this difference between genotypes increased on days 16, 27, and 42. Even after 4 months, NMII was readily detectable by qPCR in the lungs of three out of four *Myd88*^−/−^ mice, indicating long term pulmonary persistence. NMII spread systemically to all organs analyzed here and was detectable on day 7 after intratracheal infection by qPCR in decreasing quantity in liver, spleen and heart of *Myd88*^+/−^ (Figure 5). Of interest, *Myd88*^−/−^ mice harbored a substantially higher bacterial load in spleen, liver and the heart. In fact, while NMII was almost completely cleared from these organs in *Myd88*^+/−^ mice already on day 27, it was still readily detectable on d42 in most *Myd88*^−/−^ mice. However, in contrast to the continued persistence in *Myd88*^−/−^ lungs after 4 months, at this late time point NMII appeared to be eliminated from spleen, liver, and heart, based on negative qPCR results. Together, the results from the intratracheal infection experiments corroborated the findings made after intraperitoneal infection, and showed that MyD88 signaling is essential for local control of NMII in the lung after intratracheal infection, preventing systemic dissemination and initiating clearance of NMII from organ tissues.

### Decreased Granulomatous Inflammation in Myd88^−/−^ Mice After NMII Infection

**Figure 6:**
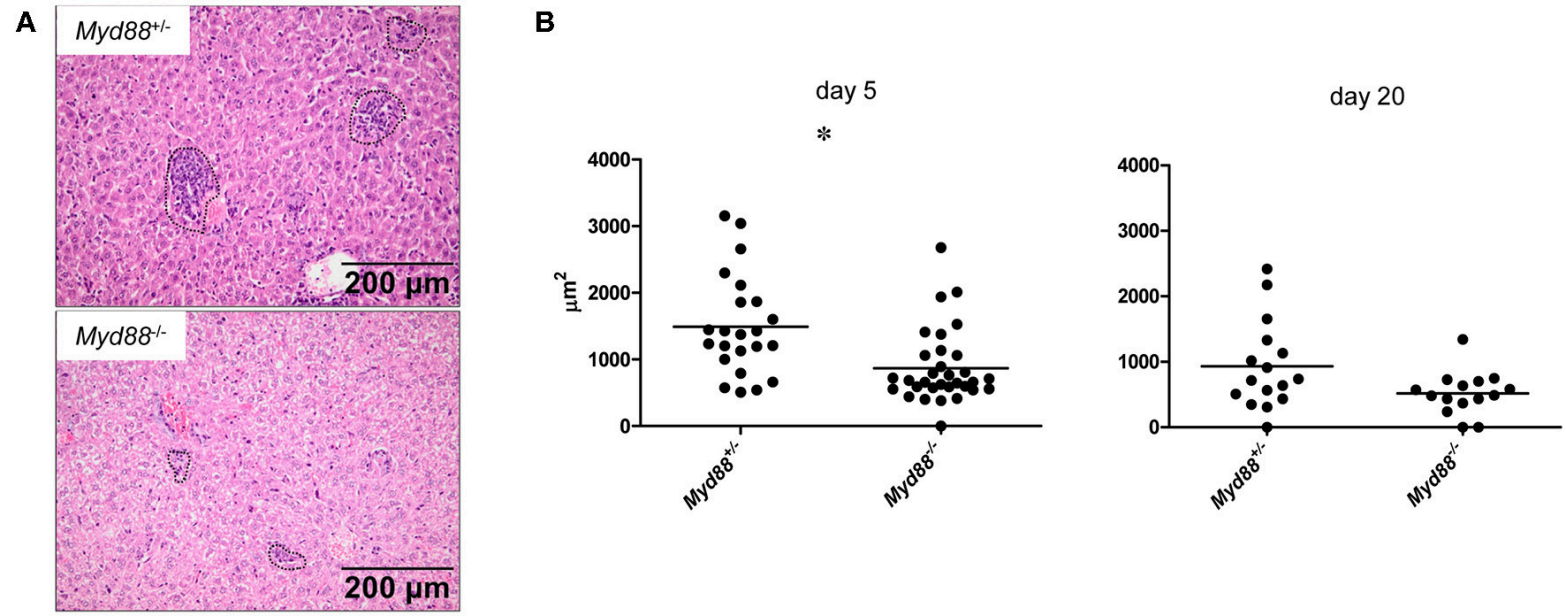
MyD88-dependent granulomatous inflammation in livers of *C. burnetii*-infected mice. *Myd88*^+/−^ and *Myd88*^−/−^ mice were infected i.p. with 5 × 10^7^ CFU NMII. H&E stained liver sections were analyzed for leukocyte infiltrates by histopathology (magnification 100x). **(A)** Granuloma-like structures were marked (indicated by dashed lines in photomicrographs, shown are examples from day 5 post infection) and the total area of all marked granulomas per field of view was measured (ZEN software, Zeiss). Eight fields of view were analyzed per individual mouse **(B)**. Results were analyzed using an unpaired two-tailed Mann-Whitney U test. (d5: *n* = 3 for *Myd88*^+/−^, *n* = 4 for *Myd88*^−/−^; d20: *n* = 2 for both genotypes).

Despite the increased and prolonged presence of NMII in the organ tissues, *Myd88*^−/−^ mice did not develop signs of clinical disease and in fact were protected from the transient weight loss induced early after infection in *Myd88*^+/−^ mice (Figure 3). To investigate the impact of MyD88 signaling on leukocyte infiltration and tissue damage, we next performed histopathological analyses of livers obtained on day 5 and 20 after intraperitoneal infection (Figure 6). In the livers of *Myd88*^+/−^ mice granuloma-like structures consisting of infiltrated leukocytes were easily detectable on day 5 and to a lesser extent on day 20 after infection, reflecting the massive reduction in bacterial load at the later time point in *Myd88*^+/−^ mice (Figure 4). In contrast, the granuloma size appeared smaller in *Myd88*^−/−^ livers. Indeed, quantitative assessment of the area occupied by granulomatous inflammation showed a reduced response in the absence of MyD88 at both time points. Thus, despite a more than 1.5-log higher bacterial burden (Figure 4), *Myd88*^−/−^ mice generated only a weak granulomatous reaction to NMII in the liver, indicating that MyD88 signaling is essential to attract leukocytes to the sites of infection. Therefore, we next turned to analyze the expression of chemokines and cytokines involved in leukocyte recruitment and in the organization of granulomatous responses.

### MyD88-Deficiency Thwarts Expression of Chemokines and of the IFNγ Response in NMII-infected Mice

**Figure 7:**
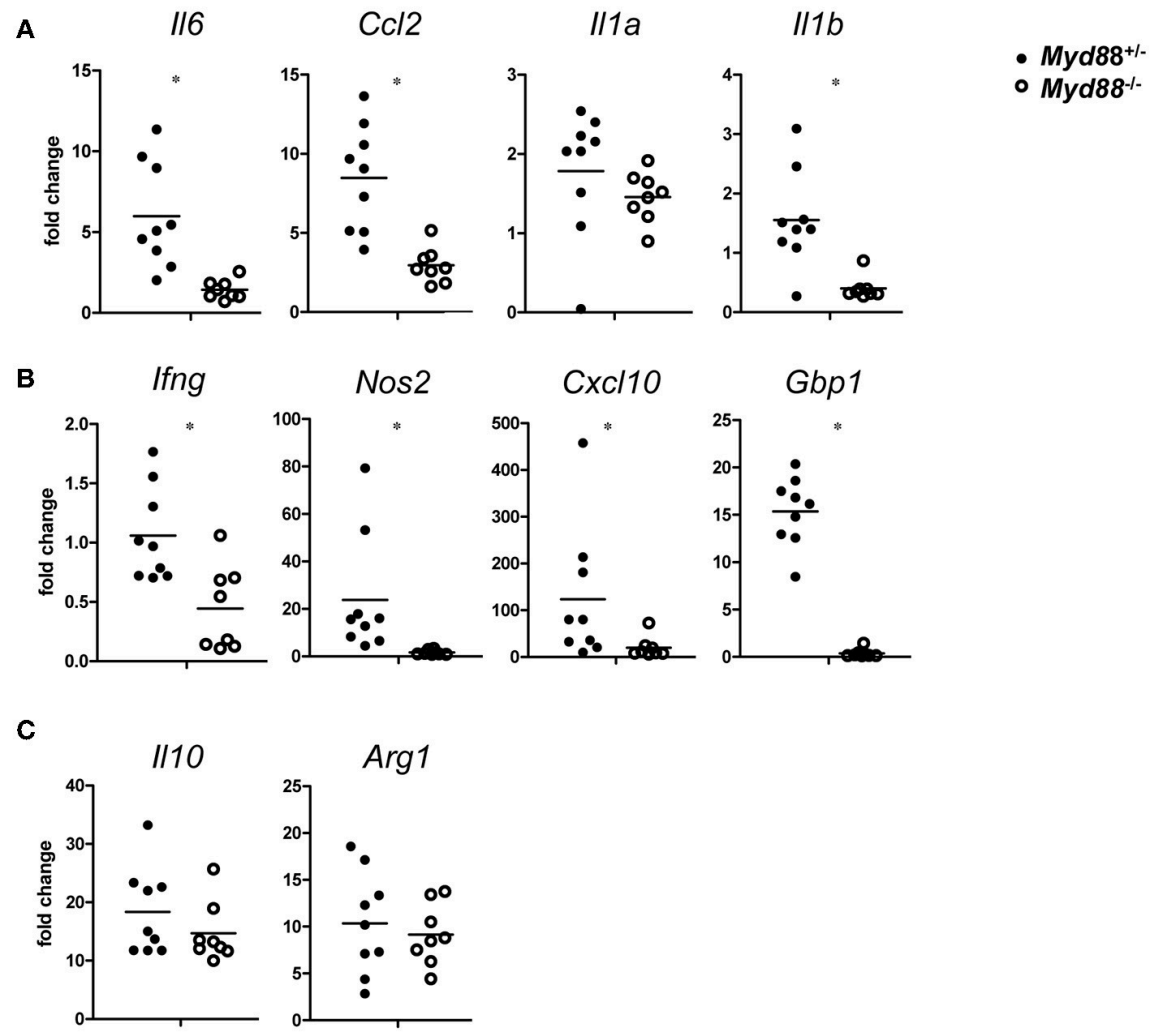
MyD88-dependence of gene expression changes in *C. burnetii*-infected mice. On day 5 after i.p. infection with NMII (5 × 10^7^ CFU), RNA from spleen was harvested and analyzed by qRT-PCR. Fold changes were calibrated to non-infected *Myd88*^+/−^ mice. Each dot represents one mouse. Data were pooled from two independent experiments. **(A)** expression of the pro-inflammatory cytokines *Il6, Ccl2, Il1a*, and *Il1b*. **(B)** expression of *Ifng* and its target genes *Nos2, Cxcl10*, and *Gbp1*. **(C)** expression of *Il10* and *Arg1*. Results were analyzed using an unpaired two-tailed Mann–Whitney *U-*test.

The chemokine CCL2 (= MCP-1) attracts CCR2^+^ monocytes to sites of infection. Expression of *Ccl2* was induced on day 5 after NMII infection and showed a clear dependence on MyD88. A similar pattern was found for the pleiotropic cytokine IL-6 and for IL-1a and IL-1b (Figure 7A). The findings of a significant reduction of CCL2 and IL-6 in *Myd88*^−/−^ mice in response to NMII infection are consistent with the observed reduction in leukocyte infiltration and granuloma response. IFNγ is pivotal for the control of *C. burnetii* replication *in vivo* (3). Its expression was reduced in a *Myd88*^−/−^ mice after infection with NMII (Figure 7B). Among the plethora of IFNγ-induced genes, iNOS stands out as an essential mediator of protection against intracellular infection in general and is required for control of *C. burnetii* replication *in vivo* in particular. MyD88-deficiency strongly impaired the induction of *Nos2* expression in infected mice (Figure 7B). An even more pronounced dependence on MyD88 signaling was observed for upregulation of *Gbp1*, a member of the IFNγ-induced p65 GTPase family (Figure 7B). The chemokine CXCL10 (= IP-10) is strongly induced by IFNγ in combination with TLR signals, and was also dependent on MyD88 signaling in NMII-infected mice (Figure 7B). In contrast to the clear MyD88-dependence of these chemokines and IFNγ-response genes, the induction of the immunoregulatory cytokine IL-10 and of the enzyme Arginase-1 by NMII was largely independent of MyD88 (Figure 7C), which contrasts to the results obtained with BMM after infection with NMII *in vitro* (Figure 2). Together, MyD88 appears to be differentially required *in vivo* for the expression of inflammatory genes and of the IFNγ response, but not for genes associated with alternative activation of macrophages such as *Il10* and *Arg1*.

## Discussion

This manuscript demonstrates an essential role for the TLR/IL-1R-associated adapter protein MyD88 in the control of *C. burnetii* replication in macrophages *in vitro* and for efficient local containment and elimination of the bacteria after infection *in vivo*. In the absence of MyD88, the expression and production of important inflammatory cytokines, chemokines and anti-microbial effector molecules was severely impaired. These data on host gene expression after infection provide clues about MyD88-dependent candidate mechanisms involved in immunological control of *C. burnetii*. Our findings are consistent with previous studies using TLR2- and MyD88-deficient mice (12, 15, 27) and with the association of a polymorphism in human *Myd88* with the development of chronic Q fever (11). Our data also show that infection of *Myd88*^−/−^ mice with the attenuated NMII strain provides a model for prolonged and systemic infection with *C. burnetii*, facilitating *in vivo* studies of the host-*C. burnetii* interaction and the resolution of inflammation.

Since MyD88 is a central adapter protein not only of TLR signaling, but also of the receptors for IL-1, IL-18, and IL-33, the impaired control of NMII in *Myd88*^−/−^ mice may be due to the combined lack of pathogen sensing through TLR and signaling through IL-1 family cytokines. While the role of IL-18 and IL-33 in *C. burnetii* infection has not been investigated yet, mice deficient in the IL-1 receptor had a transiently increased bacterial burden in the lung, but not in spleen, after intranasal infection (28), suggesting that IL-1R signaling accounts for only a minor part of MyD88's protective function. The strongly decreased induction of IL-1 expression observed by us in the absence of MyD88 (Figure 7) is consistent with the notion that its function as adapter of TLRs upstream of IL-1 and other TLR-induced cytokines is critical to initiate protective immunity.

An involvement of TLR2 in innate immune sensing of *C. burnetii* was already shown using macrophages *in vitro* (12). However, *Tlr2*^−/−^ mice were able to clear *C. burnetii* after intraperitoneal infection comparable to wild-type mice (27). More recently, Ramstead et al. demonstrated a requirement for TLR2 in protection against *C. burnetii* infection in mice after intratracheal, but not intraperitoneal infection (15). The same study also demonstrated that MyD88-deficient mice have a more dramatic increase in bacterial burden than *Tlr2* knockouts after intratracheal infection with virulent *C. burnetii* Nine Mile phase I (15). While our results corroborate the importance of intact MyD88-dependent signaling reported after infection with *C. burnetii* Nine Mile phase I using the attenuated NMII strain, it is important to point out the additional differences between both studies: first, we infected *Myd88*^−/−^ mice by both the intraperitoneal and the intratracheal route, showing that a robust increase in bacterial load was observed independent of the route of infection; second, we performed a much longer kinetic sampling that showed the prolonged presence of NMII in the lung and other organs; third, we included a comprehensive analysis of host gene expression changes after infection.

Our results indicate that deficiency in MyD88 impairs control of NMII at the level of innate as well as adaptive immunity. First, the lack of robust production of cytokines like TNF by macrophages infected with NMII is sufficient to explain the unrestricted replication *in vitro* (Figure 1), as recently demonstrated in TNF-deficient macrophages (14), and likely contributes to the increased bacterial burden during the early phase of infection *in vivo*. Impaired expression of TNF and of chemokines like CCL2 in MyD88-deficient tissues allows only a weak granulomatous response (Figure 6) with reduced production of IFNγ, likely by T cells, and in consequence suboptimal expression of anti-microbial effectors (Figure 7). Among these, iNOS is established as important contributor of protection against *C. burnetii* (4). Expression of *Gbp1*, a member of the 65 kDa family of IFNγ-induced GTPases, was abrogated in NMII-infected *Myd88*^−/−^ mice (Figure 7). Gbp1 is recruited to the phagosomes of macrophages infected with mycobacteria and is required for control of mycobacterial replication in mice (29). Whether Gbp1, and other members of this family, is essential for inhibition of replication of *C. burnetii* will be important to test in future experiments. Of note, not all host response genes to NMII infection were MyD88-dependent (Figures 2, 7). The induction of iNOS in macrophages *in vitro*, and the expression of Arginase-1 and of IL-10 *in vivo*, was not altered in MyD88-deficient cells/mice after infection with NMII. These findings demonstrate that most likely additional pattern recognition receptors or adapter protein pathways are triggered by *C. burnetii*, which may differ between infected cell types *in vitro* and *in vivo*.

Regardless of the mechanism by which MyD88 counteracts the spread and replication of NMII, the relevance of the findings in the mouse model for the course of human Q fever disease is indicated by a recent investigation of genetic variation in pattern recognition receptors and adapter proteins in patients with chronic Q fever and controls with previous exposure to *C. burnetii*, which identified a SNP in the *Myd88* promoter (-938C>A) with susceptibility to develop chronic Q fever (11). The robust differences in bacterial loads observed here in *Myd88*^−/−^ mice, indicate that infection with NMII can be successfully employed to model chronicity of Q fever. In fact, the increase in bacterial burden after intratracheal infection was considerably more pronounced than that observed in the only described model for chronic Q fever to date, the macIL-10tg mouse line overexpressing IL-10 from macrophages, after infection with the virulent *C. burnetii* Nine Mile phase I strain (10). In this context, it is also important to note that *C. burnetii* infection models in macIL-10tg and *Myd88*^−/−^ mice lead to prolonged bacterial persistence in the organs through different mechanism: first, deficient sensing of *Coxiella*-derived PAMPs through TLR signaling (*Myd88*^−/−^ mice) and, second, STAT3-dependent deactivation of TLR-induced inflammatory responses and reprogramming of macrophages (in macIL-10tg mice). Importantly, both models represent different pathophysiological mechanisms apparently operating in patients with chronic Q fever (7, 11). The finding that the attenuated *C. burnetii* strain used by us causes persistent infection in *Myd88*^−/−^ mice is in line with the recent appreciation of the NMII strain as causing lethal infection in immunocompromised SCID mice, showing that virulence of *C. burnetii* cannot be reduced to phase I LPS alone (22, 23). In contrast to SCID mice, that completely lack adaptive immunity, the *Myd88*^−/−^ mouse model has the advantage of a less severely compromised immune system and therefore much better mimics the situation in humans.

## Materials and Methods

### Generation of Bone Marrow Derived Macrophages

All mice utilized in this study were at least 6 weeks of age. C57BL/6 wildtype mice were obtained from Charles River Breeding Laboratories (Sulzfeld, Germany). The MyD88-deficient mice were generated by Dr. S. Akira (30), backcrossed to C57BL/6, and bred at the Präklinische Experimentelle Tierzentrum of the University Hospital Erlangen.

Primary bone marrow derived macrophages (BMM) from femurs and tibiae were propagated by culture in petri dishes for 7 d in complete Dulbecco's modified Eagle medium (DMEM) (Life Technologies) containing 10% fetal bovine serum (FBS) (Biochrome), antibiotics, and 50 μM β-mercaptoethanol (complete DMEM [cDMEM]) plus 10% L929 cell-conditioned medium as a source of macrophage colony-stimulating factor (M-CSF) at 37°C, 10% CO2 and 21% O2. Adherent macrophages were harvested by Accutase (Sigma, Deisenhofen, Germany) treatment, washed, and counted.

### Culture of Coxiella Burnetii

An isolate of the *C. burnetii* Nine Mile phase II strain clone 4 (NMII, RSA493) was generously provided by Matteo Bonazzi (Institut de Recherche en Infectiologie de Montpellier, Montpellier, France). One aliquot of purified *C. burnetii* NMII was propagated in a 75 cm^2^ tissue culture flask containing 30 ml acidified citrate cysteine medium (ACCM-2, 4700-003, Sunrise Science Products, San Diego, CA, USA) in an atmosphere of 37°C, 5% CO_2_ and 2.5% O_2_ (31). Following 4 days of culture, *C. burnetii* were transferred overnight to room temperature and ambient atmosphere. Subsequently, bacteria were pelleted for 30 min at 4500 x g, resuspended in 1 ml phosphate buffered saline (PBS) and quantified by optical density at OD_600_, where an OD_600_ of 1 equals ~1 × 10^9^ *C. burnetii* per ml, as determined by serial dilution, plating on ACCM-D agarose and counting of CFU after 9 days of culture.

### *In vitro* Infection of BMM

2 h prior infection, BMM were harvested, seeded and cultured at 37°C, 5% CO_2_ in RPMI complete medium containing 10% fetal calf serum (FCS), 1% HEPES and 0.5% β-mercaptoethanol (RPMI-CM). For microscopic analysis, 0.5 × 106 BMM were seeded on 10 mm coverslips in 24-well dishes, whereas 12-well dishes and a density of 1.0 × 10^6^ cells/well were used for qPCR and cytokine ELISA studies. The cells were washed with PBS and infected with *C. burnetii* at MOI 10. Following infection, cells were centrifuged for 5 min at 250 x g and incubated at 37°C and 5% CO_2_. After 4 h, cells were washed with PBS to remove extracellular *C. burnetii* and supplied with RPMI-CM.

### Quantification of Bacterial Load

We defined the ratio of *C. burnetii* genomic copies to BMM genomic copies as bacterial load per cell. *C. burnetii* genomes were quantified from isopropanol-precipitated DNA samples performing quantitative real-time PCR (qPCR) with a primer set specific for *dotA* gene as described elsewhere (32). BMM genomic copies were quantified from the same sample using a primer set specific for murine *albumin* gene (exon 7): forward, 5′-GGCAACAGACCTGACCAAAG-3′ and reverse, 5′-CAGCAACCAAGGAGAGCTTG-3′. Isopropanol-purified DNA from 10^8^ BMM was diluted 10-fold down to 10 and served as a template to generate standard curves. qPCR was carried out in 384-well optical plates on an ABI Prism 7900HT sequence-detection system (life technologies) with 5 × EvaGreen QPCR MixII-ROX (Bio&Sell, Fürth, Germany), a 0.1 μM final concentration of each primer and 2 μl isolated DNA (pediluted 1:20 in ultrapure H_2_O) as template in a final volume of 10 μl per reaction.

### Cytokine ELISA

Cytokine production of *C. burnetii* infected BMM was assessed from supernatants (SN). SNs were collected, centrifuged at 500 × g, 4°C for 10 min, transferred to a new 1.5 ml reaction tube and stored at −80°C until they were analyzed. Enzyme-linked immunosorbent assay (ELISA) kits (BDTM, Heidelberg, Germany) specific for the indicated cytokine were applied according to the manufacturer's protocol.

### Immunofluorescence

For indirect immunofluorescence microscopy analyses, *C. burnetii* infected BMM were cultured on 10 mm coverslips in 24-well dishes. At indicated points of time, cells were washed three times with equilibrated PBS, fixed for 15 min with equilibrated 4% paraformaldehyde (PFA) and permeabilized with ice-cold methanol for 1 min. Cells were quenched and blocked with 50 mM NH_4_Cl in PBS/ 5% goat serum (GS) for 60 min at room temperature. Incubation with primary antibody dilution in PBS/ 5% GS was conducted at room temperature for 60 min. Subsequently, cells were washed three times with PBS and further incubated with secondary antibodies in PBS/ 5% GS for 30 min at room temperature. After final 3 x washing with PBS, coverslips were mounted using ProLong Diamond containing DAPI. For visualization, a Carl Zeiss LSM 700 Laser Scan Confocal Microscope and the ZEN2009 software (Jena, Germany) were used.

In this study, we used primary antibodies directed against *C. burnetii* and LAMP-1 (Developmental Studies Hybridoma Bank, Iowa, IA, USA). Secondary antibodies were Alexa Fluor labeled (Alexa Fluor 488/ green, 594/ red) and purchased from Dianova, Hamburg, Germany.

### *In vivo* Infection Experiments

All mouse experiments were approved by the regional government (Regierung von Unterfranken, animal protocol 54-2532.1-44/13). Six to twelve week old *Myd88*^+/+^, *Myd88*^+/−^ and *Myd88*^−/−^ mice (on C57BL/6 background) were bred in the Präklinische Experimentelle Tierzentrum (University Hospital Erlangen) and housed in ventilated ISOcages under S2 conditions. Sex matched groups of mice were infected with *C. burnetii* either intraperitoneally (5 × 10^7^ CFU/200 μl PBS/mouse) or intratracheally (10^6^ CFU/50μl PBS/mouse). Intratracheal infection was performed by direct injection of bacterial suspension into the trachea of the mice. To do this, mice were anesthetized with isofluorane, placed in dorsal position and the trachea was accessed after incision of the skin. Prior to surgery buprenorphine (0.1 mg/kg) was injected intraperitoneally for analgesia. The physical condition of the mice was monitored regularly. At the indicated time points, mice were humanely killed and organ tissue (~20–50 mg) of spleen, liver, lung, heart was collected. Tissue samples were preserved in PeqDirectLysis® buffer (Peqlab) + Proteinase K (Roche) for DNA preparation or RNAlater® (Qiagen) for subsequent RNA isolation. For histopathology, organ pieces were fixed in PFA 4%.

### DNA Isolation and Quantification of *C. burnetii* Burden Via qPCR

Organ tissue was lysed in PeqDirectLysis® buffer + Proteinase K at 56°C under shaking overnight. Then, Proteinase K was inactivated at 85°C. DNA from organ tissue then directly was used for qPCR. To amplify *C. burnetii* DNA, a TaqMan-based quantitative PCR for the insertion sequence IS1111 was performed using 5′-CATCGTTCCCGGCAGTT-3′ as forward, 5′-TAATCCCCAACAACACCTCCTTA-3′ as reverse primer, and the internal fluorogenic probe 6FAM-CGAATGTTGTCGAGGGACCAACCCAATAAA-BBQ (TibMolbiol, Berlin, Germany). To quantitate *C. burnetii* genome equivalents, a standard curve using titrated DNA prepared from a defined number of *in vitro* cultured *C. burnetii* NMII was included in each experiment. A separate qPCR run was performed in parallel for mouse *albumin* gene copies, using titrated DNA prepared from mouse splenocytes for standard curve generation. *C. burnetii* burden per cell was calculated by normalizing bacterial genome copy numbers to *albumin* copy numbers.

### RNA Isolation and Gene Expression Analysis by qRT-PCR

RNA from organ tissue (stored in RNAlater® stabilizing reagent until further processing) was isolated using peqGold TriFastTM (Peqlab) and cDNA was synthesized using High Capacity cDNA Reverse Transcription Kit (Applied Biosystems). Primers and probes were chosen from the Universal ProbeLibrary (Roche). The fold change in gene expression was determined by the ΔΔCT method, using HPRT as housekeeping gene for calculation of ΔCT values and samples of naïve *Myd88*^+/−^ mice as calibrators.

### Processing of Organ Tissue for Histopathological Analysis

After fixation of organ pieces for 24 h in PFA 4% at 4°C tissue was washed with PBS and subsequently embedded in paraffin. Five micrometers of paraffin-embedded tissue slides were transferred to Star Frost® microscope slides for staining of the tissue.

### H&E Staining of Organ Tissue

Paraffin-embedded tissue sections were stained with Hematoxilin and Eosin (H&E) to visualize alterations in tissue structure and tissue-infiltrating leukocytes. Leukocyte infiltrates were marked by a blinded investigator and the area of each granuloma-like structure was calculated using ZEN software (Zeiss).

### Immunohistochemistry Staining of Organ Tissue

*C. burnetii* was detected using the polyclonal α-*C. burnetii* antiserum described above. Detection of bound antibodies was performed with biotinylated secondary goat ant-rabbit antibody and ABC-Kit using the peroxidase substrate method DABImmPact (all from Vector Laboratories, Burlingame, CA, USA). Quantification of *C. burnetii* positive spots in eight randomly chosen fields of view in the tissue sections was done at 100x magnification using ZEN software (Zeiss).

### Statistical Analysis

Statistical analysis was performed as described individually for each graph in the figure legends using either GraphPad Prism (version 5) or SigmaPlot. *P* < 0.05 were statistically significant and are indicated by an asterisk (^*^).

## Author Contributions

LK, IH, CD, JS-L, and BB performed experiments. LK, IH, CD, JS-L, AL, and RL designed and analyzed experiments. RL wrote the manuscript with input from LK, IH, CD, JS-L, and AL.

### Conflict of Interest Statement

The authors declare that the research was conducted in the absence of any commercial or financial relationships that could be construed as a potential conflict of interest.

## Abbreviations

TLR: Toll-like receptor
NMII: *C. burnetii* Nine Mile phase II
GE: Genome equivalents.
